# Prognostic Information on Progression to Dementia: Quantification of the Impact on Quality of Life

**DOI:** 10.3233/JAD-231037

**Published:** 2024-02-13

**Authors:** Robin Jeanna Vermeulen, Bram Roudijk, Tim Martin Govers, Maroeska Mariet Rovers, Marcel Gerardus Maria Olde Rikkert, Ben Franciscus Martinus Wijnen

**Affiliations:** a Department of Medical Imaging, Radboud University Medical Centre, Nijmegen, The Netherlands; b EuroQol Research Foundation, Rotterdam, The Netherlands; c Department of Geriatrics, Radboudumc Alzheimer Centre, Radboud University Medical Centre, Nijmegen, The Netherlands; d Center for Economic Evaluations, Trimbos Institute, Utrecht, The Netherlands

**Keywords:** Alzheimer’s disease, dementia, health state utility, mild cognitive impairment, prognosis, quality of life, risk assessment

## Abstract

**Background:**

The increasing interest in early identification of people at risk of developing dementia, has led to the development of numerous models aimed at estimating the likelihood of progression from mild cognitive impairment (MCI) to dementia. It is important to study both the need for and possible outcomes related with such prediction models, including the impact of risk predictions on perceived quality of life (QoL).

**Objective:**

This study aimed to quantify the impact that receiving a risk prediction on progression from MCI to dementia has on QoL.

**Methods:**

A Discrete Choice Experiment (DCE) and Time Trade Off (TTO) study were performed. Participants completed choice tasks related to dementia prognosis while imagining having MCI. We collected DCE data by an online survey, and TTO data via videoconferencing interviews. DCE data were analyzed using a mixed multinomial logit model and were anchored to a health state utility scale using mean observed TTO valuations.

**Results:**

296 people participated in the DCE and 42 in the TTO. Moderate and high predicted dementia risks were associated with decrements in utility (–0.05 and –0.18 respectively), compared to no prognostic information. Low predicted risk was associated with an increase in utility (0.06), as well as the availability of medication or lifestyle interventions (0.05 and 0.13 respectively).

**Conclusions:**

This study shows a significant impact of dementia risk predictions on QoL and highlights the importance of caution when sharing information about expected MCI disease courses.

## INTRODUCTION

Dementia, of which Alzheimer’s disease is the most common form, is a major cause of disability among the elderly, with a considerable impact on the affected individual, their caregivers, and society [1, 2]. Currently, no disease-modifying treatment is available to prevent the progression of symptoms in people with dementia [3, 4]. Since evidence has shown that the neuropathological processes underlying dementia start years before the onset of clinical symptoms, intervention early in the disease trajectory is believed to be most promising for future therapies [5, 6]. Consequently, there is growing interest in identifying individuals at risk of developing dementia.

Mild cognitive impairment (MCI) is a cognitive condition that is associated with increased probability of developing dementia. It is characterized by a memory impairment abnormal for age, without reduced functioning in daily activities [7]. The reported rate of progression from MCI to dementia varies, depending on recruitment setting, age of participants and duration of follow-up. Though, approximately 40% of all MCI cases progress to dementia within 5 years, whereas others remain stable or revert to normal cognition [8–11]. Identifying individuals at risk of developing dementia offers an opportunity to target and test potential therapies on those who are most likely to benefit from them.

In current practice generally no individualized prognosis on the progression from MCI to dementia is given. Many studies, however, have focused on developing models to predict the development of dementia among people with MCI [12, 13]. As with the development of other health care innovations, it is important to study both the need for and possible outcomes associated with such risk prediction models, e.g., health outcomes and potential cost-effectiveness. Except for the benefit of selecting MCI cases for treatment development studies, also other consequences of early dementia prediction should be taken into account. Early identification of those at risk for example enables early clinical management, patient counseling, and advanced care planning [14]. However, early dementia prognosis could also result in psychological harm (e.g., anxiety, depression) and stigma [15]. These aspects related to prognostic information might significantly affect an individual’s perceived quality of life (QoL).

Several methods exist to express QoL, of which one involves assigning a health state utility weight to a specific health condition. This health state utility weight is measured on a scale ranging from 0 to 1, where a value of 0 corresponds to ‘death’ and a value of 1 corresponds to ‘full health’ [16]. Utilizing the health state utility scale enables a comparison of disease severity across various medical conditions and facilitates the use of QoL information in cost-effectiveness analyses that inform healthcare decision-making. With this study, we aim to quantify the impact of receiving a dementia risk prediction on perceived QoL, expressed on a health state utility scale.

## METHODS

### Valuation methods

**Fig. 1:**
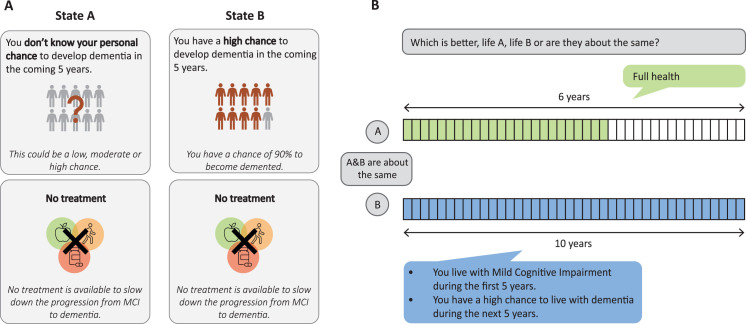
Example study tasks. (A) example DCE task; (B) example TTO task.

Health state utility values are typically based on stated preference studies, consisting of valuations by participants of the general public, who are asked to imagine to live in a specific health situation. Multiple methods exist to gather preference data [16]. In this study two health state valuation methods were used to obtain complementary data: discrete choice experiment (DCE) and time trade off (TTO) [17]. A DCE consists of relatively simple tasks in which participants are asked to choose between two scenarios that describe health conditions [18, 19]. In the remaining part of this paper, we call these scenarios health states. Answers to DCE questions elicit health state preferences and health state utilities on a latent scale, meaning that only the relative distance between health states can be determined, but no absolute health state utility value can be assigned to each of the health states. Hence, the latent scale obtained from the DCE needs to be rescaled to a health state utility scale ranging from 0 (dead) to 1 (full health). TTO, which involves more difficult, time and resource consuming tasks, yields health state utility values that can be used to anchor the DCE data to a health state utility scale [17]. In every TTO task, participants are asked to express their preference or indifference for two health episodes, namely Life A and Life B. Life A typically presents a varying period of time in ‘full health’, whereas life B presents a life of 10 years in a certain health state. The time in ‘full health’ is varied until the respondent reaches a point where he or she is indifferent between a shorter life in ‘full health’ or a longer life of 10 years in the presented health state [20]. By anchoring the DCE scale based on TTO utility values for a subset of the health states, it becomes possible to determine the health state utility value of additional health states included in the DCE. Figure 1 gives an example of a DCE and TTO task.

### Study population and data collection

People aged 60–75 from the Dutch general population were eligible to participate in this study. Two representative samples of participants were selected by I&O Research, a research agency with a respondent panel that forms a good representation of the Dutch general population [21]. Participants of the DCE were invited to participate in the study via an email, which included a link to the survey. A separate sample of respondents was selected for participation in the TTO interview. Contact details were shared by I&O Research with the researchers from the Radboudumc, who contacted the participants in order to plan the interview. All participants participated voluntarily and provided written or verbal informed consent. Data collection took place in September and October 2022. This study was granted exemption for approval by the local research ethics committee of the Radboudumc (medical research involving human subjects act) and performed according to the principles of the Declaration of Helsinki.

### Health states

Health states in this study were composed of several attributes that could vary along multiple levels. The selection and description of attributes and levels to be included, was based on an iterative process involving literature search and consultations with experts in the field (in total 3 experts in stated preference studies and 2 clinical experts). The two attributes and corresponding levels of which health states in this study were composed, are: 1) “dementia risk prediction” with four possible levels, i.e., low, moderate, or high predicted risk on progression from MCI to any type of dementia, or no personalized risk prediction; and 2) “treatment”, with three possible levels, i.e., no treatment, hypothetical medication, or lifestyle interventions. We assumed these two attributes to be of major influence on the preferences for receiving a risk prediction.

**Fig. 2:**
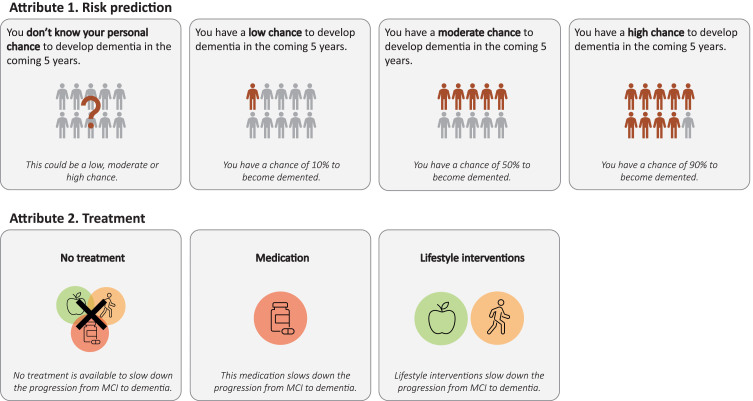
Attributes and levels included in the study.

The first attribute was included since we were specifically interested in evaluating the impact of different possible risk prediction outcomes on QoL. Levels included for this attribute were based on current practice and potential outcomes of published models for MCI to dementia risk prediction. The potential predictions were categorized in the four levels based on consultations with experts in the field. In addition, we aimed to identify the potential effect of the absence or presence of treatment on perceived QoL, when receiving a prediction on progression from MCI to dementia. For the latter we included the option ‘medication’ since in the future there might be medication available for people with MCI. The option ‘lifestyle interventions’ was included since multiple studies are being performed to study the benefits of lifestyle interventions in terms of slowing down progression from MCI to dementia [1, 22]. Figure 2 gives an overview of the attributes and levels included in this study. By combining the levels of both attributes health states were formed, resulting in 12 possible health states.

### Study procedures

#### Discrete Choice Experiment

*Questionnaire.* The DCE consisted of an online questionnaire, that started with background information on the health conditions included, the attributes and levels, and the type of questions (including one example task). As part of the background information, participants were informed that approximately 40% of all MCI cases progress to dementia within a period of 5 years. Subsequently, participants were shown 12 choice tasks (Fig. 1A). Within each choice task participants were presented 2 of the health states. Participants were asked to indicate which health state they preferred while imagining that they lived with MCI. No opt-out option was included. The example task showed two health states including levels of both attributes that were included in this study. This choice task was used as an illustration of what the questions looked like, and to show how people could indicate their preferred option. The combination of health states shown in the example task was not included in the actual questionnaire. Illustrations were added to each attribute level to increase the understandability of the information for the online respondent panel. No information was given about the total time people would live in the health states. The choice tasks were shown to the participants in a random order. At the end of the questionnaire participants received questions regarding participant characteristics, such as age, gender and educational level. Participants were also asked to indicate, on a Likert scale ranging from 1 to 5, how strongly they wished to receive information on the expected progression to dementia in case of MCI, with 1 indicating no desire to receive such information and 5 indicating a strong preference to receive such information.

*Construction of choice sets.* Based on the 12 health states that were included in this study, 12*11 = 132 unique choice tasks could be created. A subset of 12 of these was included in the questionnaire. To include the choice tasks that provide the most information, a Bayesian efficient design was created in Ngene [23]. This design assumes likely prior distributions (e.g., beta coefficients in the regression analysis) that were derived from a pilot study among 15 participants (Supplementary Table 1). Participants in the pilot study were derived via convenience sampling among people in the researchers own environment, they were contacted via the researchers directly, had ages comparable to the participants that were eligible to participate in the DCE and TTO, and they provided their consent to participate in this study. Choice tasks included in the pilot questionnaire were also created in Ngene based on small prior distributions that were assumed to be likely by the researchers (Supplementary Table 1).

*Testing the full questionnaire.* Before distributing the (pilot) questionnaire, it was tested for understandability and applicability of the background information, figures and questions, and the length of the questionnaire (face validity). For this purpose, we distributed the questionnaire among people in our own environment who were comparable to the target population of this study (i.e., by means of convenience sampling). In addition, we asked an expert in the Dutch language to evaluate whether the questionnaire would be understandable for the general Dutch population. Based on the information obtained though both sources, we optimized the background information, question format, pictograms, and language (level B1). Content validity of the questionnaire was assessed by experts in preference-based studies and clinical experts involved in the construction of the questionnaire. The full Dutch version of the questionnaire is available from the authors on request.

**Box 1:** Health states included in the TTO questions

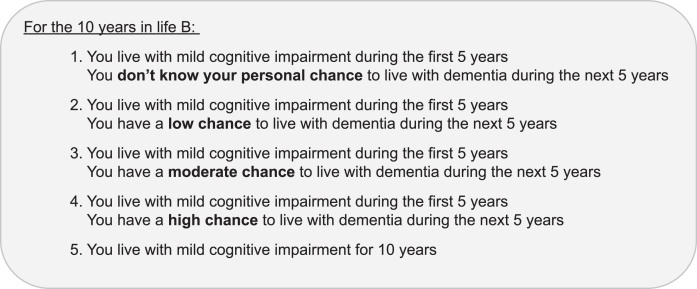

#### Time Trade Off

*Interviews.* The questions of the TTO part of this study were asked during a computer-assisted online interview. TTO produces values on a health state utility scale ranging from 0 to 1 [20]. The interviewer (RV) connected with the respondents through videoconferencing software and shared the screen to show respondents the questions and tasks to be completed. The EuroQol-Portable Valuation Technology (EQ-VT) software was used during the computer-assisted interviews, and the interviews were held using a videoconferencing program that the interviewee preferred [24]. At the start of the interview, the same background information as in the DCE regarding the health conditions and risk prediction options, including pictograms, was communicated to the respondents. This was followed by two example TTO tasks and five TTO tasks showing health states included in this study. Participants were asked to indicate between two health episodes which they preferred: one showing a life of 10 years in one of the study health states and one showing a life in full health for a time period ranging between 0 and 10 years (Fig. 1B). The time in full health was varied following a fixed iterative process until indifference between the two health episodes was reached. To reduce complexity and because not many people were expected to value the health states in this study as worse than dead, no option to obtain negative TTO values was included in this study. We made this assumption based on a meta-analysis by Landeiro et al. where both self and proxy rated utility values for dementia remain above zero [25]. The example TTO tasks consisted of situations where people for example had to imagine ‘being in a wheelchair’. The participants followed the steps of a TTO valuation task, while considering living in the presented health states, to get acquainted with the type of questions. The five TTO tasks including health states of this study were shown to the participants in a random order. At the end of the interview, the respondents also received the questions on participant characteristics and were also asked to indicate on a scale from 1 to 5 how strongly they wished to receive information on the expected progression to dementia in case of MCI.

*Construction of valuation tasks.* Construction and formulation of the health episodes included in the TTO was done in collaboration with experts in preference-based studies and clinical experts (content validity). Before starting the TTO interviews for this study, we performed pilot interviews among people in our own environment to test the understandability of the question format. Box 1 shows the description of the health episodes that were included in the TTO. The 10 years in Life B were divided into two parts: participants were asked to imagine that they would live with MCI during the first 5 years of Life B. In addition, they were informed that during the last 5 years of Life B, there was a particular chance to live with dementia. In other words, they had a predicted chance to have progressed from MCI to dementia after the first 5 years in Life B, and in case of no progression they would still be living with MCI. We also included a TTO task in which no progression risk after the first five years with MCI was included, since we were also interested in the value that people assign to living with MCI alone. Moreover, participants were told that at this moment no effective treatment is available for MCI.

### Data analysis

DCE data and the TTO data were first analyzed independently. The DCE data were used to estimate a value set on a latent scale, while the TTO data were used to anchor the DCE data on the utility scale. Analyses were performed in Stata (version 17.0) and R (version 4.1.3).

### DCE data

To check the validity of the DCE responses, a latent class analysis was performed. This analysis helps to identify classes with different preference patterns among the participants [26]. We used this analysis to identify people that completed the questionnaire with insufficient attention. For one class the coefficients were constrained to always be equal to zero, indicating that respondents in this class have no preference for each of attribute-level combinations. People with >50% chance to belong to this ‘garbage class’ were assumed to have completed the questionnaire with insufficient attention or with random responses. These participants were removed from the analysis. The number of classes in the latent class analysis was varied to determine the number of different preference groups that could be identified, with the aim to reliably remove respondents that did not give sufficient attention to the survey.

To derive the health state valuation function, DCE data were modelled using a mixed multinomial logit model. Mixed logit is the state of the art in modelling DCE data and takes into account heterogeneity in preferences by estimating parameters for each respondent at the individual level [27, 28]. This leads to more reliable estimates than other models such as the conditional logit, that do not take heterogeneity of preferences into account. The utility function of the model takes the following equation:

$$\begin{array}{c} U_{\mathrm{ij}}=\beta_{2i}{\mathrm{LowRiskpred}}_{\mathrm{ij}}+\beta_{3i}{\mathrm{ModerateRiskpred}}_{\mathrm{ij}}\\ \;\;\;\;\;\;\;+\beta_{4i}{\mathrm{HighRiskpred}}_{\mathrm{ij}}+\beta_{5i}{\mathrm{Medication}}_{\mathrm{ij}}\\ \;\;\;\;\;\;\;+\beta_{6i}{\mathrm{Lifestyle}}_{\mathrm{ij}}+{ɛ}_{\mathrm{ij}} \end{array}$$

where *U*_*ij*_ represents the observable relative score of participant i for choice set j, β_2_–β_6_ are coefficients of the attributes indicating the relative weight placed on the attributes, and *ni* represents the standard deviation of the random parameter for each respondent *i*. Finally, *nij* + *ɛij* captures the individual-specific unexplained variance around the mean. The health state ‘no personalized risk prediction + no treatment available’ was taken as the reference health state in the evaluation of the DCE data.

### TTO data

Health state utility values per health state were first determined per person. This was done by dividing the time in ‘full health’, that was valued as being equal to a longer life of 10 years in one of the health states, by 10. Subsequently, mean utility values per health state were determined.

### DCE and TTO data combined

The assumption was made that participants interpreted the four TTO tasks including risk prediction in a similar way as the DCE health states consisting of risk prediction levels without availability of treatment. To anchor the DCE data to the health state utility scale, the mean observed TTO values were mapped on the predicted latent DCE values for these states. A linear regression was fitted through this combined data:

$$U_{\mathrm{TTO}}=\beta_{0}+\beta_{1}*U_{\mathrm{DCE}}$$

where β_0_ (= intercept) and β_1_ (= slope) are the rescaling parameters. By multiplying the coefficients of the mixed multinomial logit model with the value of β_1_, rescaled health state utility values were obtained.

### Subgroup analysis

We performed two subgroup analyses to assess whether differences exist in the impact on QoL depending on the stated wish for receiving a prediction and the experience with dementia through people in the environment. For these analyses the DCE sample was divided in subgroups. The mixed multinomial logit model was run for each of the subgroups and these were anchored to the health state utility scale based on the total TTO sample. Regarding wish for prediction the DCE sample was divided in three groups: a group that wished to receive a risk prediction (score 4 and 5), a group that was neutral (score 3) and a group that did not want to receive such a prediction (score 1 and 2). The second subgroup analysis consisted of a subgroup of people who had experience with dementia in their environment and a group that did not have experience with dementia in their environment.

## RESULTS

Table 1 shows the demographic characteristics of the participants, also in comparison with the Dutch population aged 60 to 75 years [29]. A total of 296 participants completed the online DCE questionnaire. Latent class analysis showed that up to three different preference classes (plus garbage class) could be distinguished. Thus, a model with 4 classes best fitted the data. Answers from 3.7% of the cases were flagged to be of suspicious quality, according to a likelihood of > 50% to belong to the garbage class. This resulted in inclusion of 285 participants in the analysis. The TTO interview was completed by 42 participants. One participant accidently participated in both the DCE and TTO. This person was removed from the analysis of the TTO data, since this was the second part this person was involved in, resulting in 41 participants in the TTO analysis. In both the DCE and TTO there was an oversampling of higher educated people.

**Table 1:** Respondent characteristics of the DCE and TTO samples

Characteristic	DCE sample, *n* = 285	TTO sample, *n* = 41	Dutch general population aged 60–75
Gender % (*n*)
Female	49.5 (141)	53.7 (22)	50.7
Male	50.5 (144)	46.3 (19)	49.3
Other	0 (0)	0 (0)	–
Age distribution % (*n*)
60–64	54.7 (156)	19.5 (8)	35.5
65–69	25.3 (72)	43.9 (18)	31
70–75	20 (57)	36.6 (15)	33.5
Age, *mean (sd)*	65.4 (4.3)	67.9 (4.2)	–
Education % (*n*)
Low	6.0 (17)	7.3 (3)	28.65
Lower middle	45.6 (130)	17.1 (7)	37.59
Upper middle	34.7 (99)	51.2 (21)	23.89
University/post graduate	13.7 (39)	19.5 (8)	9.10
Unknown	–	4.9 (2)	–
Valuation own health (1–7)^*^
*Mean (sd)*	5.3 (1)	5.6 (1.1)	–
MCI experience^†^ % (*n*)
Yes	69.8 (199)	61 (25)	–
No	30.2 (86)	34.1 (14)
Unknown	–	4.9 (2)
Dementia experience^†^ % (*n*)
Yes	73.0 (208)	75.6 (31)	–
No	27.0 (77)	24.4 (10)

^*^Overall health was valued on a Likert scale where 1 corresponded to ‘very bad’ and 7 corresponded to ‘perfect’. ^†^People were asked whether they have experience with these conditions in their personal environment, e.g., via work, family, or friends.

### DCE data

Table 2 (column 1) shows the coefficients as a result of modelling the DCE data using mixed logit when ‘no personalized risk prediction + no treatment available’ is taken as the reference state. Table 2 (column 2) shows the rescaled coefficients, after anchoring the DCE data to the health state utility scale. The largest negative weight is assigned to a high predicted risk on progression to dementia. The largest positive weight is assigned to the availability of lifestyle interventions to slow down the progression from MCI to dementia.

**Table 2:** Modelling results of the DCE data

Variable	Mixed logit	Mixed logit anchored
	LogOdds (sd)	Rescaled coefficients on
		utility scale, mean (sd)
Intercept *(No personalized prediction; No treatment)*	–	0.75
*Low Risk Predicted*	+0.77 (0.02)	+0.06 (0.00)
*Moderate Risk Predicted*	–0.63 (0.67)	–0.05 (0.05)
*High Risk Predicted*	–2.31 (3.93)	–0.18 (0.31)
*Medication*	+0.60 (0.19)	+0.05 (0.02)
*Lifestyle interventions*	+1.68 (0.17)	+0.13 (0.01)

Rescaling constant β_0_ (= intercept): 0.75; rescaling factor β1 (= slope): 0.08.

### TTO data

Table 3 summarizes the mean observed TTO values for the health episodes consisting of a combination of MCI with each of the four different risk prediction levels and for MCI alone. Supplementary Figure 1 shows the distribution of the valuation for all TTO responses. Only one person preferred to die immediately instead of living with MCI for 10 years, which indicates that this person values MCI as worse than dead. In six of the 164 other TTO tasks a participant preferred to die immediately over a longer life of 10 years with MCI and a predicted chance to progress to dementia.

**Table 3:** Mean observed TTO values

Health state	TTO utility value
	Mean (sd)
MCI	0.85 (0.20)
MCI + no personalized risk prediction	0.77 (0.21)
MCI + low predicted conversion risk	0.8 (0.20)
MCI + moderate predicted conversion risk	0.68 (0.23)
MCI + high predicted conversion risk	0.57 (0.20)

### Anchoring: DCE and TTO data combined

**Fig. 3:**
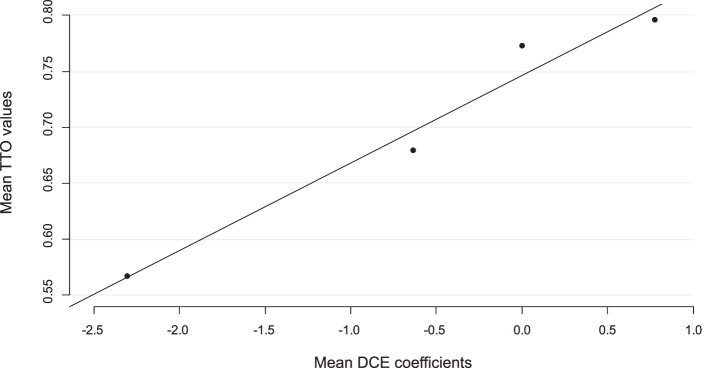
Anchoring using mean observed TTO values and predicted DCE values.

Linear mapping was used to anchor the observed TTO values on the latent DCE values, which is visualized in Fig. 3. The constant obtained with this analysis corresponds to the health state ‘MCI with no personalized risk prediction on conversion to dementia and no treatment available’ and was 0.75. The rescaling factor, which is the value of the slope in the linear regression, identified with this analysis was 0.08. All coefficients in the mixed multinomial logit model were multiplied by this factor to derive the final valuation set (Table 3). The analysis shows that, compared to no personalized risk prediction, a high predicted risk on progression to dementia was associated with the highest disutility value of 0.18, whereas the availability of lifestyle interventions to slow down progression was associated with the highest gain in utility of 0.13. Using this value set the highest utility value, 0.94, was assigned to ‘a low predicted risk on progression from MCI to dementia with the availability of lifestyle interventions’. The lowest utility, 0.57, was assigned to ‘a high predicted risk on progression to dementia without the availability of any treatment’.

### Wish for prediction

**Fig. 4:**
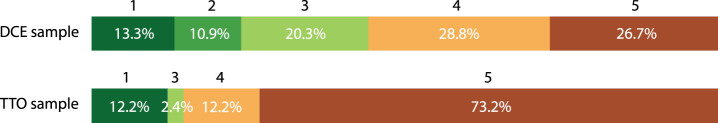
Wish for receiving a prediction on progression from MCI to dementia on a Likert Scale 1–5^*^. ^*^Likert Scale ranged from 1 to 5 : 1 = I do not want to receive prognostic information on progression to dementia, 5 = I would certainly prefer to receive prognostic information on progression to dementia.

Figure 4 shows the answers of participants when asked to indicate on a scale from 1 to 5 how strongly they preferred to receive a prediction on progression to dementia in case of MCI. In the DCE 55.5% of the participants indicated that they preferred to receive such a prediction (score 4 and 5), whereas 24.2% indicated that they did not prefer to receive a dementia risk prediction (score 1 and 2) and 20.3% indicated to be neutral (score 3). A higher percentage of 85.4% of the participants in the TTO indicated to have a preference for receiving a dementia risk prediction, whereas 12.2% did not want to receive such a prediction and 2.4% indicated to be neutral. Frequently mentioned reasons for desiring to receive a risk prediction on the progression from MCI to dementia were: the possibility to make arrangements with family, to start advanced care planning, and to have more certainty about the future.

### Subgroup analysis

Some small differences in the rescaled coefficients were found for some of the attribute levels in the subgroup analysis based on wish for prediction (Supplementary Table 2 and Supplementary Figure 2). The valuations suggest that the subgroup favoring to obtain predictive information, tends to place a higher value on the availability of treatment than the other subgroups. The second subgroup analysis showed no difference in impact on QoL for the group of people who had experience with dementia in their environment and a group that did not have experience with dementia in their environment (Supplementary Table 3 and Supplementary Figure 3).

## DISCUSSION

The results of our DCE and TTO study among people aged 60–75 years showed that most participants would prefer to receive a prognosis on progression to dementia in case of MCI. However, our results also show that a moderate or high predicted risk on progression were associated with considerable decrements in QoL compared to not receiving a personalized prognosis. Contrastingly, a low predicted risk was associated with a gain in health state utility compared to not receiving a risk prediction. Furthermore, the availability of treatment was associated with a gain in utility, with lifestyle interventions to slow down progression showing the highest gain in utility. Subgroup analyses only showed small differences for the analysis based on wish for prediction. The subgroup of respondents indicating to prefer prognostic information on progression to dementia in case of MCI, tend to place higher value on the availability of treatment than the other subgroups. This might indicate that people who favor information on progression risk, have a pronounced hope that treatment based on the predicted risk might slow down the progression to dementia.

Our results are in agreement with some other studies that show the desire of people with MCI to receive information on expected course of their symptoms [30, 31]. Frequently mentioned reasons for desiring this information, e.g., planning for the future and relief from uncertainty, were also stated by participants in our study [31]. The increase in health state utility value found for a low predicted risk on progression to dementia is in line with a study by Bartzsch et al. [32], that shows an improvement in well-being and a reduction of fears and worries in participants with a reduced risk for Alzheimer’s disease. In contrast to our findings, they found neither a significant deterioration of the mental situation nor an increase of fears and worries among participants with an elevated risk. Our results indicate that a high predicted risk on progression to dementia is associated with a threefold higher decrease in health state utility value than the increase that is associated with a low predicted risk. Landeiro et al. published a meta-analysis about self and proxy rated QoL for MCI and dementia [25]. The utility value of 0.85 for MCI obtained in our study, is comparable to the self-rated utility value of 0.86 in the study of Landeiro et al., and somewhat higher than the proxy-rated (caregiver) value of 0.80 that was found in their study. Proxy ratings of QoL for people with dementia generally are lower than those self-rated [33]. In our study people provide a proxy rating for their own QoL when living with MCI. In this valuation task they were informed that there was no risk of progression to dementia, whereas in real life there might always be a fear of progression.

The major strengths of this study are that this is the first study showing empirical data on the impact that dementia risk predictions have on QoL when measured on a health state utility scale. Furthermore, since we expressed this impact on a health state utility scale, it is possible to use this information in health economic evaluations. In cost-effectiveness analyses, which are commonly used to inform healthcare decision-making, effects are expressed in quality adjusted life years (QALYs), a measure combining both survival and QoL. In order to be used for the calculation of QALYs, QoL has to be expressed on a health state utility scale [16]. This study was also specifically designed to study the impact of dementia risk predictions on QoL, whereas often the wish for prognostic information is studied as part of a bigger study including people with a specific interest in participation, e.g., genetic testing for dementia. Fourth, we included a respondent sample in our study that matches the general population (albeit a small oversampling of higher educated participants). The good agreement between the TTO and DCE, shown by little error in the mapping, in our study ensures a reliable mapping of the study results.

Some limitations should also be taken into consideration. First, it is known that people tend to find it difficult to value probabilities in preference based studies [34]. Although this might have had an impact on the exact health state utility values found in this study, we do not expect that this would have an impact on the pattern of increases or decrements is QoL for the risk prediction levels compared to no prognostic information. Second, preference-based studies to obtain health state value sets are generally performed in the general population. It has been shown, however, that people with a certain health condition tend to adapt and value the health condition better than the general public [35]. Third, we only included two attributes in the construction of the health states. However, some other aspects might also be of influence when making the choice to receive or not to receive prognostic information, e.g., the accuracy of the risk prediction or informal caregiver situation (e.g., having children or a partner). We did consider inclusion of these attributes. Eventually, we did not include them, since we assumed the two attributes that were included in the final questionnaire to be the most important when quantifying the impact of prognostic information on quality of life. Inclusion of additional attributes might draw away the attention from the risk prediction itself, and shift the focus towards other attributes that were considered of less relevance for the current study. Moreover, when including these additional attributes, the scenarios would move further away from the concept ‘health state’, which would make the instrument less suitable for the calculation of utility values. In addition, as DCE and TTO tasks are already quite difficult to understand, we assumed that inclusion of extra attributes (especially information on accuracy) would make the questions too difficult. It could be questioned whether participants would be able to fully understand what the accuracy of a risk prediction means, since risk is a concept that is difficult for many people to comprehend [36]. In the background information, however, we explained to participants that a prediction model will not reach a 100% accuracy, and that there will be some degree of uncertainty around the prediction. Fourth, for the TTO we had a relatively small study sample, which results in a relatively big standard error and therewith uncertainty about the obtained health state utility values. Fifth, this study included people from the Dutch general population. Effects could be different in other countries, as previous studies have shown that preferences and utility values corresponding to health states differ between countries [37, 38].

### Conclusion

The last decades focus has shifted towards identification of people at risk for developing dementia earlier in the disease process, which has led to the development of models to predict MCI to dementia conversion. This study shows that prognostic information is associated with considerable changes in QoL. The decrease in QoL after a high predicted risk is threefold higher than the increase in QoL after a low predicted risk. This shows that we should be cautious when it comes to sharing information on the expected course of complaints to people with MCI. Since most of the respondents indicated to wish to receive such prognostic information this also underlines the importance to carefully inform about possible advantages and disadvantages of prognostic testing. One should strive for a shared decision making between patient and doctor before starting prognostic testing and sharing associated information.

## Supplementary Material

Supplementary Material

## Data Availability

The data supporting the findings of this study are available on request from the corresponding author.
