# Supplementary material for: Prognostic Information on Progression to Dementia: Quantification of the Impact on Quality of Life
Source: J Alzheimers Dis. 2024 Feb 13;97(4):1829–40. doi: 10.3233/JAD-231037 (PMC10894563; doi:10.3233/JAD-231037)

Supplementary Material

Prognostic Information on Progression to Dementia: Quantification of the Impact on Quality of Life

Supplementary Table 1. Prior distributions for creating DCE choice sets in the pilot and final questionnaire.*

**Attribute**	**Assumed prior values for pilot questionnaire**	**Prior values based on pilot**
No personalized prediction	0	- 0.67
Moderate predicted conversion risk	- 0.1	- 1.06
High predicted conversion risk	- 0.2	- 2.26
Medication	+ 0.1	+ 1.30
Lifestyle interventions	+ 0.1	+ 1.94

*reference state: low predicted risk and no treatment

Supplementary Table 2. Rescaled DCE coefficients for the total group and subgroups based on wish for prediction.

**Variable**	**Total group N = 285** Mean (sd)	**Subgroup 1 N = 69** Mean (sd)	**Subgroup 2 N = 58** Mean (sd)	**Subgroup 3 N = 158** Mean (sd)
Intercept *(No personalized prediction; No treatment)*	0.75	0.76	0.75	0.72
Low Risk Predicted	+ 0.06 (0.00)	+ 0.04 (0.03)	+ 0.05 (0.03)	+ 0.09 (0.01)
Moderate Risk Predicted	- 0.05 (0.05)	- 0.08 (0.03)	- 0.05 (0.17)	- 0.02 (0.01)
High Risk Predicted	- 0.18 (0.31)	- 0.20 (0.19)	- 0.19 (0.41)	- 0.15 (0.03)
Medication	+ 0.05 (0.02)	- 0.01 (0.16)	- 0.01 (0.09)	+ 0.10 (0.04)
Lifestyle interventions	+ 0.13 (0.01)	+ 0.08 (0.05)	+ 0.08 (0.03)	+ 0.18 (0.05)

Subgroup 1 (score 1 and 2 on Likert scale) = no preference for receiving a dementia risk prediction; subgroup 2 (score 3) = neutral regarding the wish for receiving a dementia risk prediction; subgroup 3 (score 4 and 5) = preference to receive a dementia risk prediction.

Supplementary Table 3. Rescaled DCE coefficients for the total group and subgroups based on experience with dementia in the environment.

**Variable**	**Total group N = 285** Mean (sd)	**Subgroup 1 N = 208** Mean (sd)	**Subgroup 2 N = 77** Mean (sd)
Intercept *(No personalized prediction; No treatment)*	0.75	0.75	0.75
Low Risk Predicted	+ 0.06 (0.00)	+ 0.06 (0.01)	+ 0.06 (0.01)
Moderate Risk Predicted	- 0.05 (0.05)	- 0.05 (0.01)	- 0.06 (0.08)
High Risk Predicted	- 0.18 (0.31)	- 0.18 (0.23)	- 0.18 (0.28)
Medication	+ 0.05 (0.02)	+ 0.05 (0.01)	- 0.06 (0.03)
Lifestyle interventions	+ 0.13 (0.01)	+ 0.15 (0.03)	+ 0.11 (0.03)

Subgroup 1 = people that have experience with dementia through people in their environment; subgroup 2 = people that do not have experience with dementia through people in their environment.

Supplementary Figure 1. Distribution of TTO responses**.**

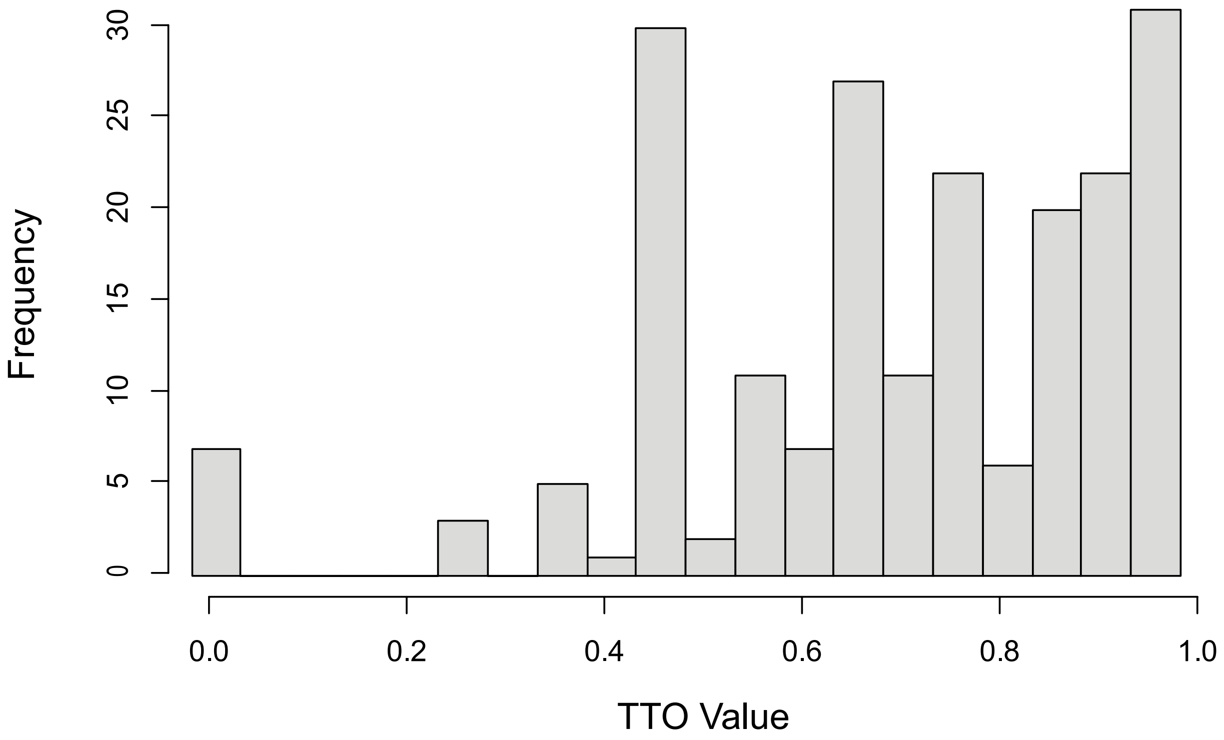

Supplementary Figure 2. Mean rescaled DCE coefficients for subgroups based on wish for prediction.

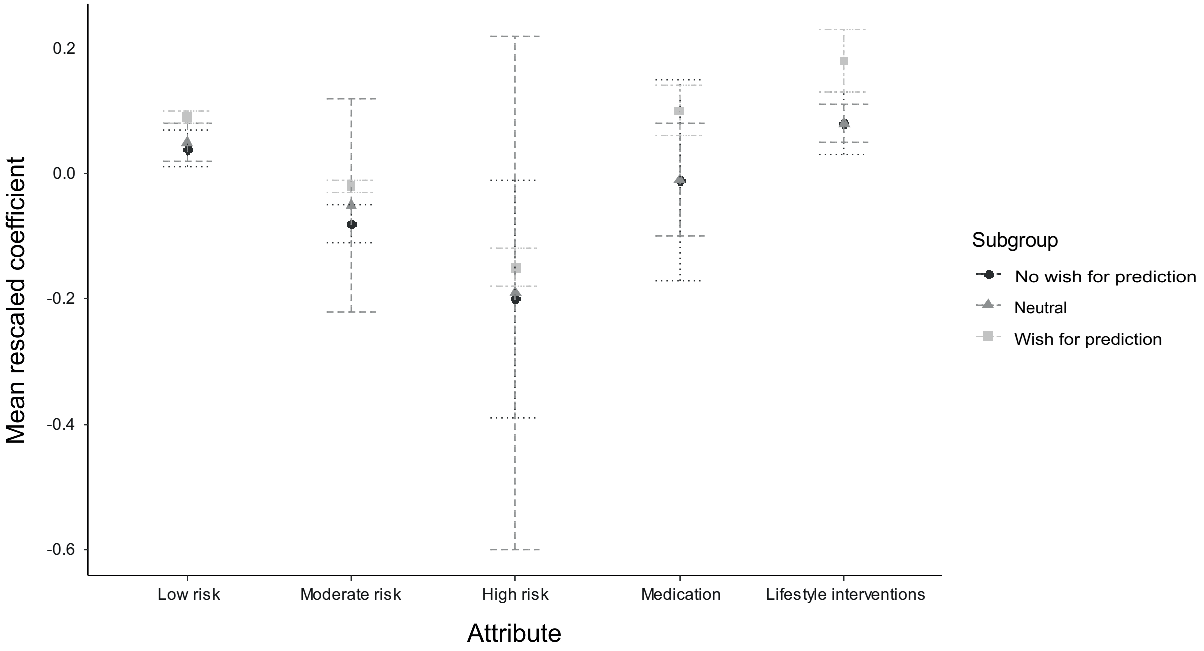

Supplementary Figure 3. Mean rescaled DCE coefficients for subgroups based on experience with dementia in the environment.

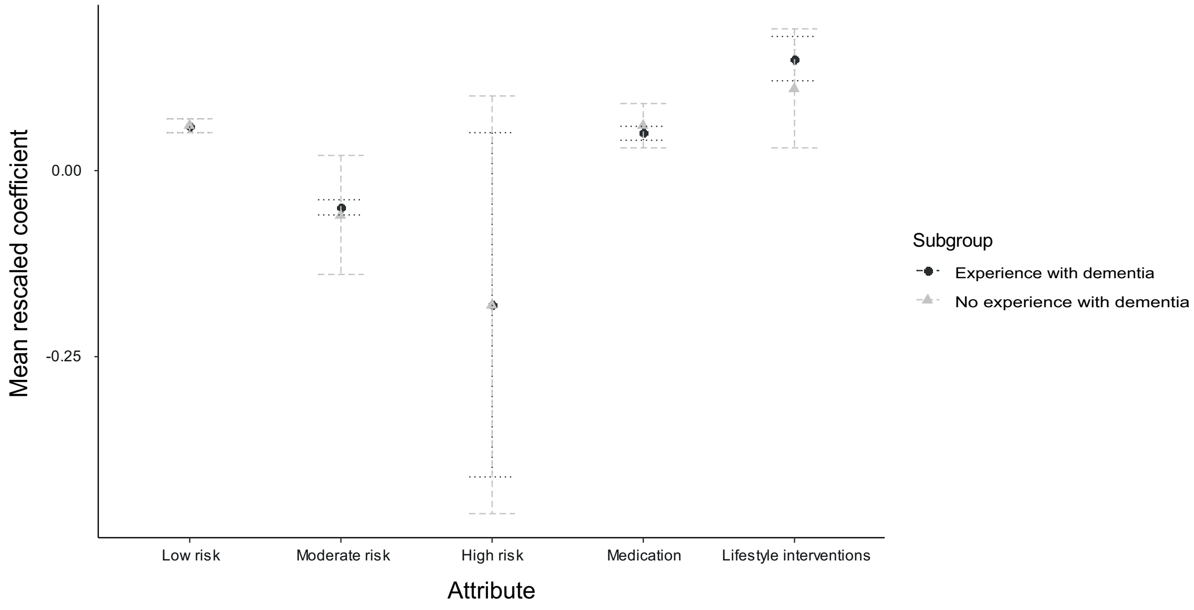

Supplement: Supplementary Material [file jad-97-jad231037-s001.pdf]
